# A nomogram to predict prognosis of patients with unresected hepatocellular carcinoma undergoing radiotherapy: a population-based study

**DOI:** 10.7150/jca.30365

**Published:** 2019-07-25

**Authors:** Li Zhang, Li Yan, Hao Niu, Jie Ma, Bao-Ying Yuan, Yu-Han Chen, Yuan Zhuang, Yong Hu, Zhao-Chong Zeng, Zuo-Lin Xiang

**Affiliations:** 1 Department of Radiation Oncology, Zhongshan Hospital, Fudan University, 180 Feng Lin Road, Shanghai 200032, China; 2 Department of Radiation Oncology, Eye & ENT Hospital, Fudan University, Shanghai, 200031, China; 3 Department of Radiation Oncology, Shanghai East Hospital, Tongji University School of Medicine, 150 Jimo Road, Shanghai 200120, China

**Keywords:** hepatocellular carcinoma, radiotherapy, prognosis, nomogram

## Abstract

**Background:** Radiotherapy is a primary treatment strategy for patients with unresectable hepatocellular carcinoma (HCC); however, the prognostic factors among HCC patients who have received radiotherapy but not undergone surgery have not been systematically studied. Thus, the prognostic factors were investigated in this study based on the Surveillance, Epidemiology, and End Results (SEER) Medicare database.

**Methods:** A screening process was used for select cases from the SEER database. Survival was analyzed using the Kaplan-Meier method and log-rank test, the Cox proportional hazards regression model, and a competing risk model. A nomogram was established for predicting 1- and 3-year overall survival (OS) of patients.

**Results:** A total of 1305 HCC patients who received radiotherapy but had not undergone surgery were included in this study and divided into training (n = 1175) and validation cohorts (n = 130). Patients in the training cohort had a 1-year OS rate of 30.9±1.3%, a 3-year OS rate of 10.0±1.0%, and a median survival rate of 6.0 months (range, 5.4-6.6 months). Race (*p* = 0.025), T stage (*p* < 0.001), N stage (*p* < 0.001), M stage (*p* < 0.001), and chemotherapy (*p* < 0.001) were identified as independent risk factors by multivariate analyses in the training cohort, while sex, age, grade, marital status, and insurance status were not independent factors. Survival in patients who received radiotherapy was worse with respect to the following characteristics: black race; higher T, N, or M stage; and no chemotherapy. A nomogram was established based on the results of the multivariate analysis, which was internally validated by a concordance index (C-index) of 0.731±0.016 and a group of calibration plots. External validation was carried out and the C-index was 0.738±0.049, which demonstrated the effectiveness of the nomogram we constructed.

**Conclusions:** Race, T stage, N stage, M stage, and chemotherapy were independent risk factors for survival of HCC patients who received radiotherapy but had not undergone surgery. A validated nomogram was formulated to predict 1- and 3-year OS in these patients based on individual clinical characteristics.

## Introduction

As the most common pathologic type of primary liver cancer and the sixth most prevalent cancer worldwide, hepatocellular cancer (HCC) are frequent with 782,500 new cases each year and the incidence of deaths is on the rise^1^, ^2^. Most patients with HCC are diagnosed at an intermediate-to-advanced stage^3^. Less than 20 % of patients with HCC are eligible for potentially curative surgical resection or liver transplantation therapy due to multi-centric tumors, vascular invasion, extrahepatic metastases, or other co-morbidities^4^. Radiotherapy is a primary and useful strategy for such patients with unresectable HCC; however, there are few reports with a focus on prognosis among a large sample of unresectable HCC patients who have received radiotherapy.

A nomogram represents as intuitive graphical solution of a statistical predictive model and generates a numerical probability of a clinical event, which has been widely used in predicting survival in cancer patients^5^; however, a nomogram that predicts overall survival (OS) in HCC patients who have received radiotherapy but have not undergone surgery does not exist. To fully identify the predictive factors which influenced the prognosis of HCC patients treated by radiotherapy alone, we analyzed medical records from the Surveillance, Epidemiology, and End Results (SEER) database (http://seer.cancer.gov/) and developed a nomogram to visually predict the prognosis of these patients.

## Methods

### Ethics statement

The Ethics Committee of the Zhongshan Hospital, Fudan University exempted the study, because the SEER is a public database and no personal information is included.

### Study patients and design

**Figure 1:**
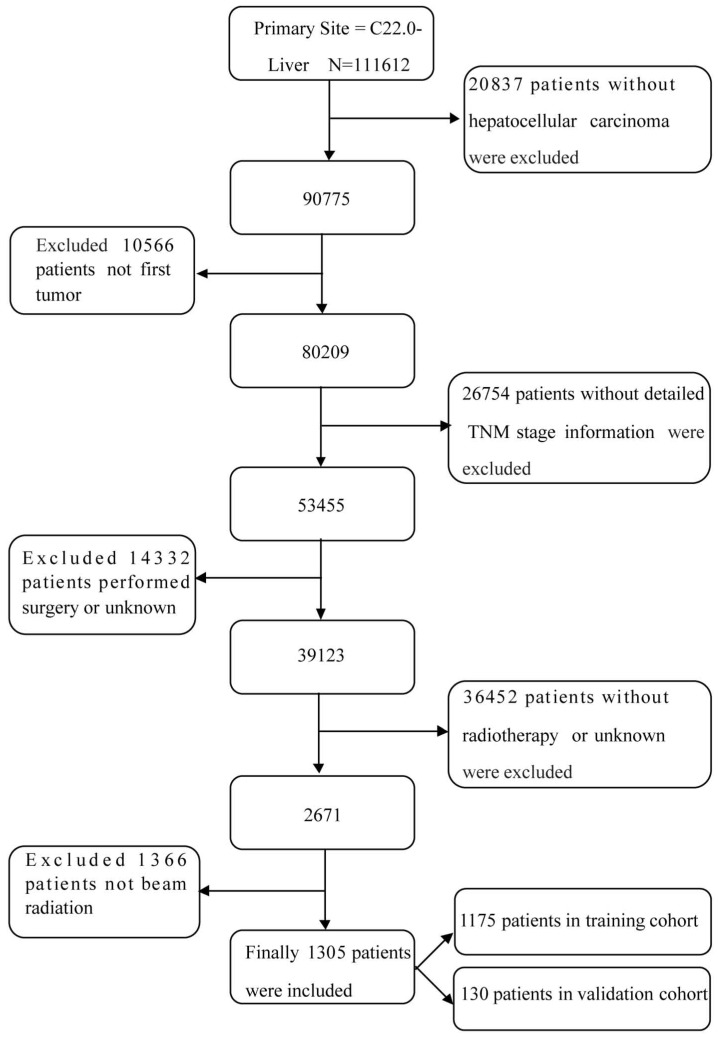
The flow diagram of the selection process for the study.

SEER (Incidence - SEER 18 Regs Custom Data with additional treatment fields, Nov 2016 Sub, 1973 - 2014 varying) data were obtained via the SEER*Stat software (version 8.3.5; http://seer.cancer.gov/seerstat/). To acquire sufficient data from the database, the selection process is shown in Figure 1. Briefly, patients with labeled primary sites C22.0-Liver were carefully reviewed. The inclusion criteria were as follows: (1) no history of other malignancies before HCC diagnosis; (2) a positive pathologic diagnosis; (3) received radiation, but had not undergone surgery; and (4) follow-up data is available. The exclusion criteria were as follows: (1) without HCC; (2) not first tumor; (3) without detailed TNM stage information; (4) performed surgery or unknown; (5) without radiotherapy or unknown; (6) not beam radiation. Finally, cases diagnosed before 2004 were excluded because there was no stage information of them. And OS was from that date of diagnosis to the date of death.

For each patient, we collected relevant sociodemographic data (race, sex, and age), final pathologic analysis (histologic type and grade, and T, N, and M stages), therapy (radiotherapy and chemotherapy), and follow-up data (cause of death, HCC-specific death, and survival time) from the SEER database. Ninety percent of patients were randomly divided into the training cohort and 10% of patients were classified into the validation cohort to externally validate the nomogram.

### Statistical analysis

In the training cohort, survival curves for each clinicopathologic factor were depicted using the Kaplan-Meier method and compared using the log-rank test. Then, the significant variables were validated by competing risk analysis and subsequently analyzed by the Cox regression model for multivariate analysis.

A nomogram was built on the basis of the results of multivariate analysis. Bootstrap resampling was used for validation of the nomogram and calibration curve construction, as previously reported^6^, ^7^. The performance of the nomogram was internally measured by the concordance index (C-index) and assessed by comparing the nomogram-predicted probability with the observed probability. Then, the nomogram was further validated by comparing the nomogram-predicted probability of the patients in the validation cohort with actual survival.

Statistical analyses were carried out using SPSS 24.0 (IBM Corp., Armonk, NY, USA) and the package of ggplot, rms of R 3.4.3 (https://www.r-project.org/). All statistical tests were 2-tailed and a *p* < 0.05 was considered statistically significant.

## Results

### Patient characteristics

As shown in Figure 1, a total of 1305 HCC patients who had received radiotherapy but had not undergone surgery were included according to the screening criteria mentioned above between 2004 and 2014; 1175 patients were randomly divided into the training cohort and 130 patients were divided into the validation cohort. As shown in Table 1, most of the patients in the training cohort had the following characteristics: white race (71.3%); male gender (82.9%); 51-60 years of age (36.5%); Gx (73.2%); and stage IV (65.1%). Of the patients, 44.9% had received chemotherapy, approximately 50% of the patients were married, and 75.8% of the patients were insured at the time of diagnosis.

### Survival analysis

**Figure 2:**
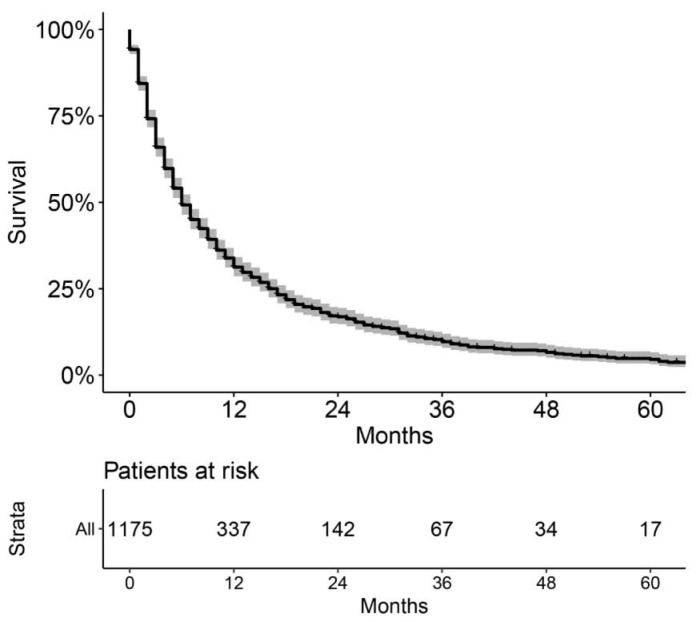
Overall Kaplan-Meier survival curve of the included patients in training cohort.

**Figure 3:**
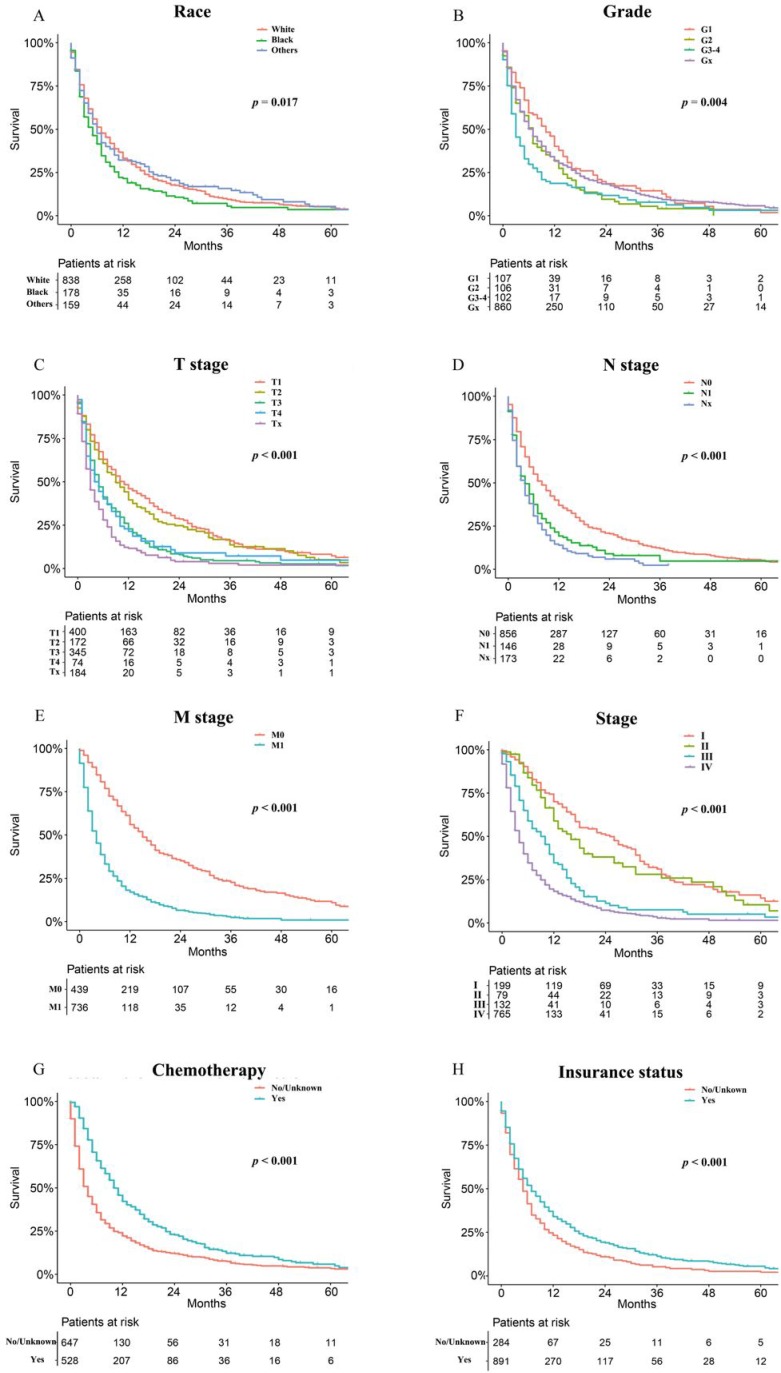
Overall Kaplan-Meier survival curves for patients in training cohort according to (A) Race, (B) Grade, (C) T stage, (D) N stage, (E) M stage, (F) Stage, (G) Chemotherapy, (H) Insurance status.

**Figure 4:**
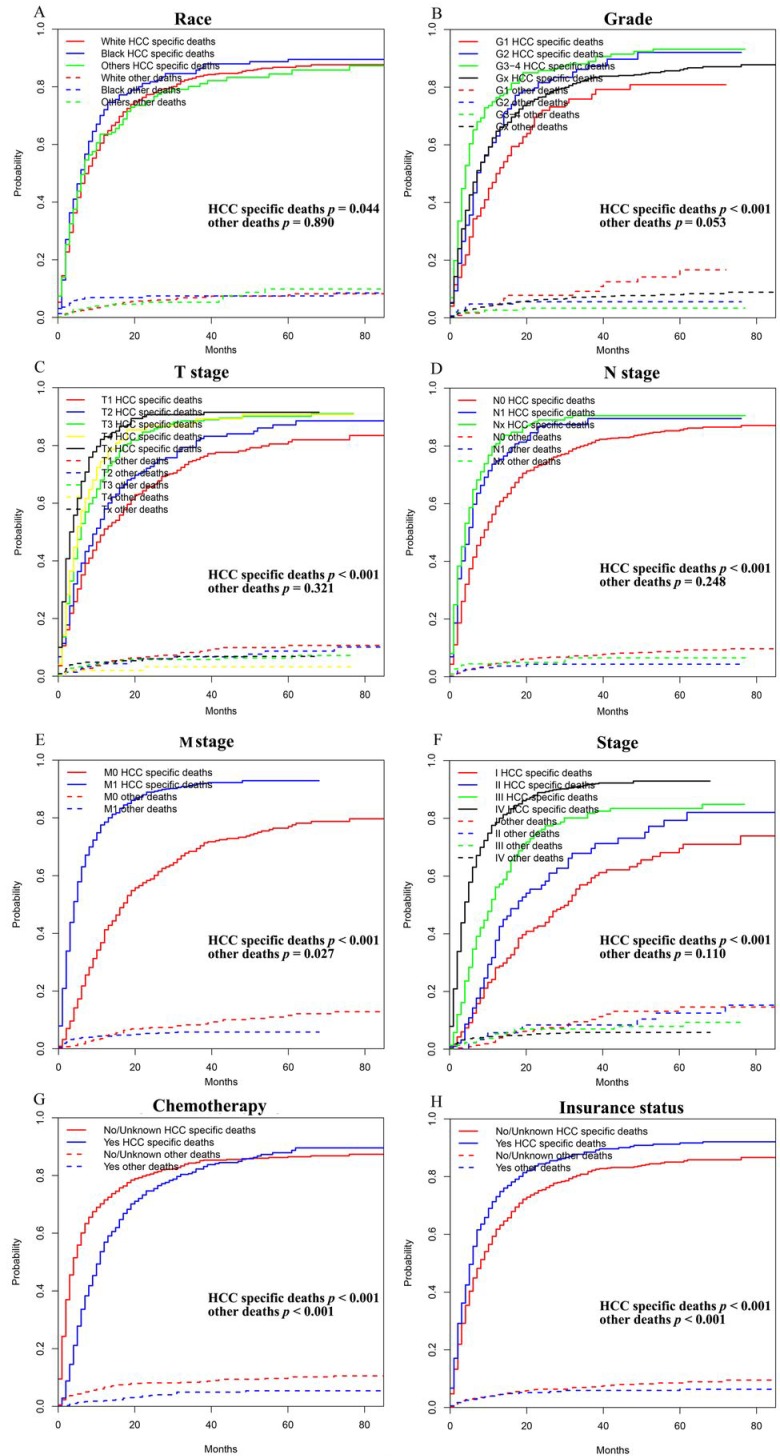
Competing risk analyses for patients in training cohort according to (A) Race, (B) Grade, (C) T stage, (D) N stage, (E) M stage, (F) Stage, (G) Chemotherapy, (H) Insurance status.

As shown in Figure 2, the patients in the training cohort had a 1-year OS of 30.9±1.3%, a 3-year OS of 10.0±1.0%, and a median survival of 6.0 months (range, 5.4-6.6 months). Univariate analyses revealed that race (*p* = 0.017), pathologic grade (*p* = 0.004), T stage (*p* < 0.001), N stage (*p* < 0.001), M stage (*p* < 0.001), stage (*p* < 0.001), chemotherapy (*p* < 0.001), and insurance status (*p* < 0.001) were significant prognostic factors, as shown in Figure 3 and Supplementary Figure 1, while sex (*p* = 0.260), age (*p* = 0.101), and marital status (*p* = 0.475) were not significantly correlated with OS. The HCC-specific survival curves based on the competing risk model were summarized in Figure 4, and show consistent results compared to the univariate analysis.

The significant factors were further analyzed in multivariate analysis. The results showed that race(*p* = 0.025), T stage (*p* < 0.001), N stage (*p* < 0.001), M stage (*p* < 0.001), and chemotherapy (*p* < 0.001) were independent prognosis factors, while grade (*p* = 0.255) and insurance (*p* = 0.634) were not considered as independent risk factors (Table 1). The variable stage was not included in the multivariate analysis because the variable stage was not independent from the T, N, and M stages. As the Odds Ratio(OR) values in Table 1 show, black race, higher T, N, or M stage, and no chemotherapy were associated with a worse survival.

### Construction and validation of the nomogram

**Figure 5:**
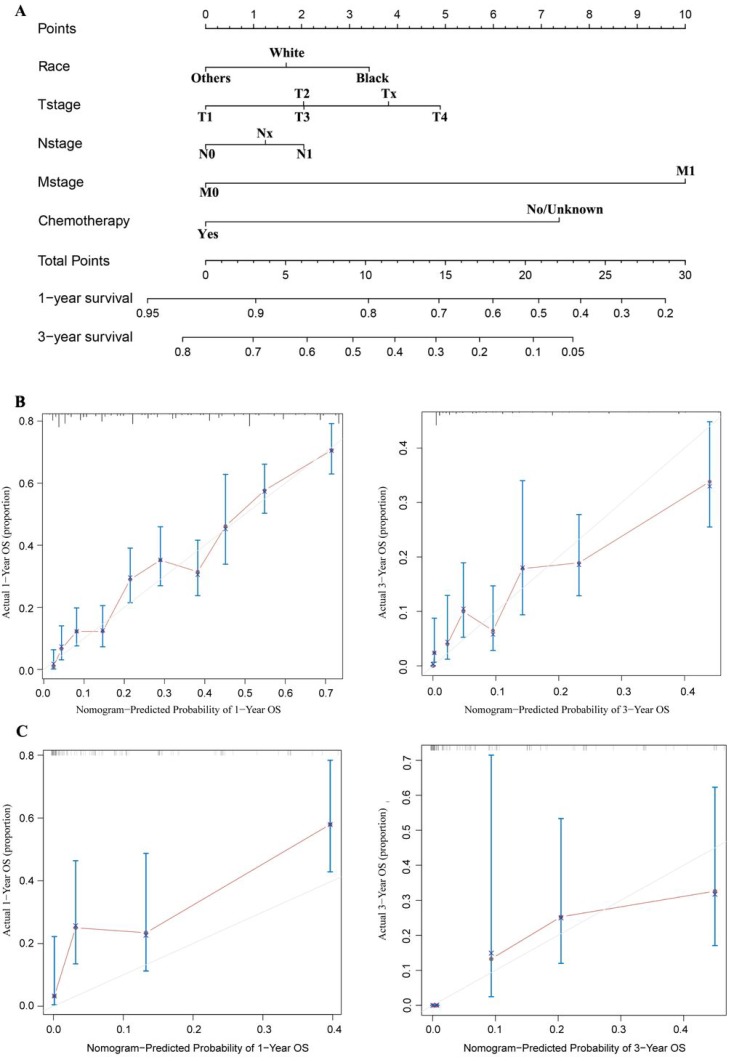
A nomogram for prediction of 1- and 3-year OS rates of patients with unresected HCC undergoing radiotherapy (A); Calibration curve of the nomogram predicting 1-year and 3-year OS rates of unresected HCC patients undergoing radiotherapy in training cohort (B); Calibration curve of the nomogram predicting 1-year and 3-year OS rates of patients with unresected HCC undergoing radiotherapy in the validation cohort (C).

The nomogram was developed and included the following variables based on the final multivariate model, as shown in Figure 5A: race; T, N, and M stage; and chemotherapy. The calibration plots based on internal bootstrap resampling validation were illustrated in Figure 5B. The C-index for prediction of OS was 0.731±0.016, indicating that the nomogram is in good agreement with the actual observation for HCC patients who had received radiotherapy but had not undergone surgery. Furthermore, the validation cohort data were used for external validation of the nomogram. After comparing the nomogram-predicted survival with the actual survival of patients in the validation cohort, a C-index of 0.738±0.049 was obtained based on the calibration plots shown in Figure 5C. Therefore, the nomogram reliably predicted the 1- and 3-year OS rates.

## Discussion

In the current study we analyzed the risk factors for HCC patients who received radiotherapy but had not undergone surgery. Greater than 1000 patients were analyzed and we showed that race, T, N, and M stages, and chemotherapy were independent prognostic factors and established a nomogram to visually and vividly predict patient survival. A small portion of the patients were isolated for external validation to demonstrate the reliability of the nomogram. Finally, both internal and external validations demonstrated the accuracy and effectiveness of the nomogram. The nomogram, a statistical model based on a combination of significant factors, will contribute to evaluating the individualized prognosis and instructing treatment of the patients. In this way, treatment guidelines and prediction of outcomes would be more efficient and accurate for doctors and patients. With its wide range of clinical applications, the nomogram is a very reliable and available predictive system.

Most unresectable HCCs have invaded the intrahepatic large vessels or metastasized and are associated with extremely poor outcomes^8^. Approximately 30% of patients were T3 and 60% were M1 in the current study, with a 1-year OS rate of 30%. In a multicenter phase I trial, 56% of unresectable patients who received radiotherapy had a partial response and 28% showed stable disease, with a 1-year OS rate of 61%^9^. Therefore, radiotherapy is a promising, non-invasive therapeutic modality for patients with unresectable HCC. Radiotherapy may also potentially serve as a bridging therapy for patients awaiting transplantation and can be used in combination with other locoregional or systemic therapies, such as transarterial chemoembolization and sorafenib^10^-^13^.

Blacks had a poorer prognosis in our analysis and several other reports^14^,^15^, likely due to the higher rates of hepatitis C virus and hepatitis B virus infections. Sex and age were not prognostic factors in the current study, which is consistent with a previous report^16^, though younger patients and female patients usually have better survival in most types of cancer.

A number of studies have indicated that pathologic grade is a predictor of survival in patients with HCC^17^, ^18^. In the present study, pathologic grade was strongly associated with 1- and 3-year survival, but pathologic grade was not an independent prognostic factor. Poorly differentiated lesions are significantly larger in size and cannot be resected^19^. Additionally, TNM stage is known to significantly predict survival in patients with unresectable HCCs, which was also demonstrated in our study. Tumor size > 5 cm in size represents a poor prognosis for HCC and is a predictive factor for early HCC recurrence^20^, ^21^. Additionally, large tumors have a higher risk of vascular invasion and higher tumor grade^22^. HCCs with and without lymph node metastases have different survival rates (5 months vs. 12 months, respectively)^23^. Metastasis is a leading cause of early recurrence and poor prognosis in HCC patients. Patients with metastatic HCC often do not survive > 1 year^24^.

Approximately 45% of patients in our study were confirmed to have received chemotherapy concurrent with radiotherapy, and these patients had a significantly better survival. In recent years, a variety of novel chemotherapeutic agents have become available to improve patients' survival. The multi-kinase inhibitor, sorafenib, is a standard therapy for advanced HCC and is often combined with chemotherapy to increase safety and efficiency^25^, ^26^. The expression of PD-L1 has been reported to be correlated with poor survival of HCC patients who have received radiotherapy^27^. A study in a murine model showed that the combination of anti-PD-L1 and radiation significantly improved the anti-tumor effect, suggesting a novel combination strategy of immunoradiotherapy in HCC^28^.

Several previous studies have revealed HCC-specific prognostic variables. For instance, the serum level of AFP was most commonly used biomarker in HCC diagnosis and prognosis^29^. However, AFP expression in many cases of liver cancer is not elevated^30^. And the patient gender effect have potentially influenced AFP levels, which increased in women^31^. And overexpression of PARPBP correlated with tumor progression and poor prognosis in HCC patients after surgery^32^. Although such variables may be potentially useful for the prognosis prediction, the reported prognostic factors for HCC OS are different among studies^33^. Compared with the prognostic variables, nomograms can provide more personalized assessment for the prognosis of patients. Wang et al. developed a nomogram that combined BMI, tumor stage, distant metastases, HBsAg, LDH, GGT and ALB, for 3- and 5-year OS in patients with AFP-negative HCC in single institution^34^. Several published nomograms have been established for predicting prognosis after radiotherapy for brain metastases and lymph node metastasis(LNM) from HCC. Park Y et al. developed a nomogram predicting the survival of 97 patients with brain metastasis from HCC treated with whole brain radiotherapy (a C-index of 0.74). This retrospective study was conducted in a relatively small study population due to the low incidence of brain metastasis from HCC^33^. Kim Y et al. developed a nomogram to predict 2-year OS with good accuracy (a C-index of 0.72) in patients who received radiotherapy for abdominal LNM from HCC. This nomogram was based on a relatively large sample size with 228 patients through the use of a multi-institutional study. The nomogram combined the significant prognostic factors, including Child-Pugh classification, status of intrahepatic tumor, presence of distant metastasis, location and number of metastatic abdominal lymph nodes(LNs), serum level of AFP, and the LN response to radiotherapy^35^. WEE et al. Developed a nomogram for predicting 6-month survival with a C-index of 0.77 in HCC patients treated with radiotherapy for LNM. In consideration of the inclusion of both intra-abdominal and extra-abdominal LN metastasis, the LN-related symptoms are highly associated with the patients' survival, which may affect the accuracy of the prognostic model^36^. Based on the results of the present study, a nomogram was formulated using race, T, N, and M stages, and chemotherapy. We constructed the nomogram for predicting 1- and 3-OS based on the clinical information in both internal validation with C-index of 0.731±0.016 and external validation with C-index of 0.738±0.049 in unresected HCC patients undergoing radiotherapy. This is the first study that developed a nomogram for unresected HCC patients undergoing radiotherapy. Though a large number of samples, tumor markers have not been included in the nomogram.

There were several limitations in this study that should be noted. First, because the SEER database did not provide detailed information regarding radiotherapy and chemotherapy, we could not calculate the effect of radiation technology, dose, and interval, and chemotherapeutic reagents. Second, the reasons that patients did not undergo surgery were not always recorded. Some patients did not undergo surgery for reasons other than the tumor, such as hepatitis or poor liver function. Third, the patients were all from the USA, thus the results might not be applicable to other populations.

In conclusion, we showed that race, T, N, and M stage, and chemotherapy are independent risk factors for survival of HCC patients who have received radiotherapy, but have not undergone surgery. We established a nomogram to facilitate visual prediction of 1- and 3-year OS based on individual clinical characteristics. Though both internal and external validation demonstrated the reliability of the nomogram, further studies are warranted.

## Supplementary Material

Supplementary figure 1.

## Figures and Tables

**Table 1:** Patient characteristics and 1-, and 3-year OS rates

Variable	Number of patients	OR(95%CI)	1-year OS (%)	3-year OS(%)	Median survival	*p* value (univariate analysis)	*p* value (multivariate analysis)
Total cases	1175(100%)		0.309±0.013	0.100±0.010	6.0(5.4-6.6)		
**Race**						0.017	0.025
White	838(71.3%)	Reference	0.332±0.017	0.094±0.012	7.0(6.0-8.0)		
Black	178(15.1%)	1.287(1.083-1.530)	0.215±0.032	0.055±0.019	5.0(3.6-6.4)		
Other	159(13.5%)	0.982(0.814-1.184)	0.322±0.039	0.157±0.032	6.0(5.4-6.6)		
**Sex**						0.260	not included
Male	974(82.9%)	Reference	0.307±0.016	0.091±0.011	6.0(5.4-6.6)		
Female	201(17.1%)	0.908(0.769-1.074)	0.335±0.039	0.124±0.027	7.0(5.2-8.8)		
**Age(years)**						0.101	not included
0-50	112(9.5%)	Reference	0.267±0.044	0.112±0.034	5.0(3.8-6.2)		
51-60	429(36.5%)	1.132(0.899-1.425)	0.262±0.022	0.068±0.016	6.0(5.2-6.8)		
61-70	336(28.6%)	0.907(0.715-1.151)	0.352±0.028	0.143±0.023	8.0(5.9-10.1)		
71-80	221(18.8%)	1.007(0.785-1.292)	0.358±0.033	0.078±0.020	7.0(5.5-8.5)		
81+	77(6.6%)	1.060(0.770-1.460)	0.354±0.058	0.078±0.039	7.0(4.6-9.4)		
**Pathologic grade**						0.004	0.255
G1	107(9.1%)	Reference	0.401±0.051	0.144±0.039	10.0(7.5-12.5)		
G2	106(9%)	1.333(0.993-1.789)	0.319±0.047	0.041±0.022	7.0(5.8-8.2)		
G3-4	102(8.7%)	1.644(1.224-2.208)	0.187±0.040	0.078±0.029	3.0(1.9-4.1)		
Gx	860(73.2%)	1.140(0.911-1.426)	0.315±0.017	0.100±0.012	7.0(6.2-7.8)		
**T stage**						< 0.001	< 0.001
T1	400(34.0%)	Reference	0.460±0.026	0.159±0.022	11.0(8.9-13.1)		
T2	172(14.6%)	1.132(0.926-1.383)	0.396±0.039	0.136±0.031	9.0(6.2-11.8)		
T3	345(29.4%)	1.765(1.500-2.078)	0.228±0.024	0.045±0.014	5.0(4.2-5.8)		
T4	74(6.3%)	1.708(1.310-2.228)	0.215±0.049	0.072±0.033	4.0(2.1-5.9)		
Tx	184(15.7%)	2.482(2.050-3.005)	0.116±0.024	0.029±0.015	3.0(2.2-3.8)		
**N stage**						< 0.001	< 0.001
N0	856(72.9%)	Reference	0.370±0.017	0.120±0.013	8.0(6.9-9.1)		
N1	146(12.4%)	1.518(1.255-1.837)	0.192±0.034	0.048±0.023	4.0(2.5-5.5)		
Nx	173(14.7%)	1.851(1.554-2.203)	0.139±0.027	0.024±0.015	4.0(3.1-4.9)		
**M stage**						< 0.001	< 0.001
M0	439(37.4%)	Reference	0.560±0.025	0.226±0.024	15.0(12.8-17.2)		
M1	736(62.6%)	2.820(2.447-3.250)	0.171±0.014	0.024±0.007	4.0(3.6-4.4)		
**Stage**						< 0.001	not included
I	199(16.9%)	Reference	0.700±0.035	0.311±0.041	25.0(18.8-31.2)		
II	79(6.7%)	1.244(0.904-1.711)	0.589±0.059	0.281±0.059	16.0(11.5-20.5)		
III	132(11.2%)	2.253(1.728-2.936)	0.348±0.045	0.076±0.028	v		
IV	765(65.1%)	3.628(2.968-4.434)	0.183±0.015	0.029±0.007	4.0(3.6-4.4)		
**Chemotherapy**						< 0.001	< 0.001
No/Unkown	647(55.1%)	Reference	0.222±0.017	0.075±0.012	4.0(3.4-4.6)		
Yes	528(44.9%)	0.566(0.498-0.643)	0.422±0.023	0.122±0.017	10.0(8.9-11.1)		
**Marital status**						0.475	not included
Married	607(51.7%)	Reference	0.313±0.020	0.083±0.014	6.0(5.2-6.8)		
Others	568(48.3%)	1.047(0.923-1.187)	0.312±0.020	0.109±0.015	7.0(6.0-8.0)		
**Insurance status**						< 0.001	0.634
No/Unkown	284(24.2%)	Reference	0.232±0.025	0.052±0.015	5.0(4.2-5.8)		
Yes	891(75.8%)	0.759(0.659-0.874)	0.340±0.017	0.113±0.013	7.0(6.0-8.0)		

## Abbreviations

HCC: hepatocellular carcinoma
SEER: Surveillance, Epidemiology, and End Results
OS: overall survival
C-index: concordance index
OR: Odds Ratio
