# Supplementary material for: A nomogram to predict prognosis of patients with unresected hepatocellular carcinoma undergoing radiotherapy: a population-based study
Source: J Cancer. 2019 Jul 25;10(19):4564–73. doi: 10.7150/jca.30365 (PMC6746140; doi:10.7150/jca.30365)
Supplement: Supplementary file 1 — Supplementary figure 1. [file jcav10p4564s1.pdf]

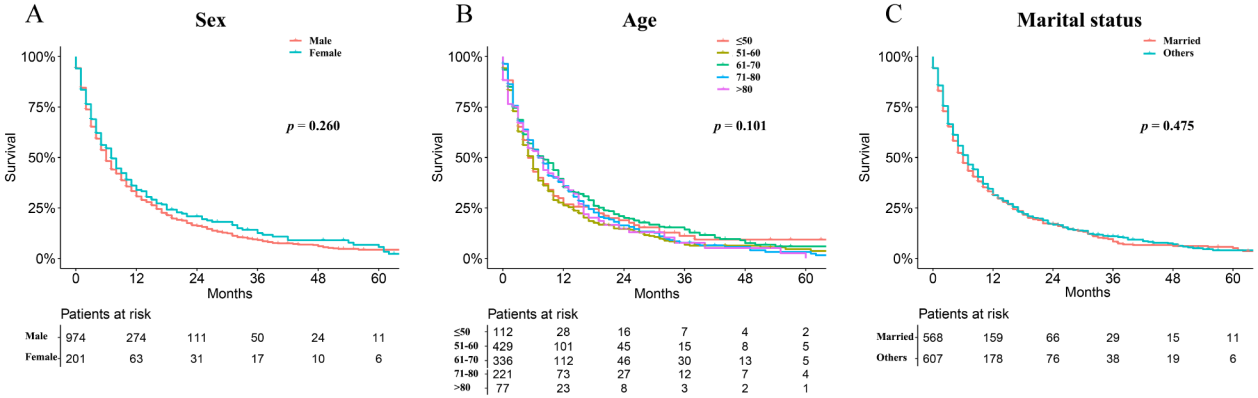

Supplementary Figure.1
